# Globalization and local response to epidemiological overlap in 21st century Ecuador

**DOI:** 10.1186/1744-8603-2-8

**Published:** 2006-05-19

**Authors:** William F Waters

**Affiliations:** 1 Institute for Research in Health and Nutrition, Universidad San Francisco de Quito, Quito, Ecuador

## Abstract

**Background:**

Third World countries are confronted by a complex overlay of two sets of health problems. Traditional maladies, including communicable diseases, malnutrition, and environmental health hazards coexist with emerging health challenges, including cardiovascular disease, cancer, and increasing levels of obesity. Using Ecuador as an example, this paper proposes a conceptual framework for linking epidemiologic overlap to emerging social structures and processes at the national and global levels.

**Discussion:**

Epidemiologic trends can be seen as part of broader processes related to globalization, but this does not imply that globalization is a monolithic force that inevitably and uniformly affects nations, communities, and households in the same manner. Rather, characteristics and forms of social organization at the subnational level can shape the way that globalization takes place. Thus, globalization has affected Ecuador in specific ways and is, at the same time, intimately related to the form in which the epidemiologic transition has transpired in that country.

**Summary:**

Ecuador is among neither the poorest nor the wealthiest countries and its situation may illuminate trends in other parts of the world.

As in other countries, insertion into the global economy has not taken place in a vacuum; rather, Ecuador has experienced unprecedented social and demographic change in the past several decades, producing profound transformation in its social structure. Examples of local represent alternatives to centralized health systems that do not effectively address the complex overlay of traditional and emerging health problems.

## Introduction: epidemiologic transition and globalization

This paper begins with the premise that global public health is not at its core only a medical issue but is, rather, embedded in social, cultural, political, and economic structures and processes. Moreover, changes in those structures and processes involve the evolution of patterns of health and wellness, which can be described in terms of epidemiologic transition and overlap. While this transition is part of broader processes related to globalization, globalization is not necessarily an essentially monolithic force that inevitably, invariably, and uniformly affects nations, communities, and households in the same manner. Rather, local specificities and forms of organization can and do shape the way that both globalization and the epidemiologic transition take place. Thus, globalization has affected Ecuador in specific ways and is, at the same time, intimately related to the form in which the epidemiologic transition has transpired in that country.

Globalization has been viewed from a variety of perspectives and is at the center of overlapping debates. One debate focuses on the fundamental nature of globalization: is it essentially a narrowly-defined economic and financial process of integration of national economies into an international economy, or does it also include more broadly-defined interweavings of political, technological, and cultural processes? This debate is framed by a broader issue: has globalization benefited most people in the world or not? A different debate concerns the relationship between globalization, public health, and the epidemiologic transition [1]. In this context, globalization affects public health in a variety of ways because it has unleashed profound changes that have redefined how institutions at many levels–nation states, government agencies, transnational corporations, multilateral organizations, non-governmental organizations, public and private health care providers, community-based and other affinity-based organizations, communities, and households–operate and interact with one another.

At the same time, the world is currently in the midst of an epidemiologic transition, defined as:

the evolutionary changes in different societal settings from a situation of high mortality, high fertility, short life expectancy, young age structure, and predominance of communicable diseases; especially in the young, to one of low mortality, low fertility, increasing life expectancy, aging, and predominance of degenerative and man-made diseases, especially among the middle and old ages [[2]: 5].

The epidemiologic transition incorporates the demographic transition (the change from high mortality and fertility to low mortality and fertility) as well as evolving patterns in the causes of morbidity and mortality. At the heart of the epidemiologic transition is a shift in the determinants of mortality and morbidity, whereby infectious and communicable diseases are supplanted by chronic and non-communicable conditions. This transformation is not uniform, however; it transpires in different ways and different times among and within different societies, and at different velocities. Thus, the transition experienced by presently industrialized countries in the past differs significantly from the experience of underdeveloped countries at present. Moreover, presently underdeveloped countries follow different patterns of transition [2-4]. As discussed below, one difference between past and present experience is that in countries like Ecuador, increasing rates of chronic and non-communicable disease associated with increasing longevity and a gradually aging population are experienced by continued high levels of infectious and communicable disease. Moreover, as discussed below, patterns of morbidity and mortality differ among socioeconomic groups due in large part to differences in their relationship to globalizing forces.

While the global reach of economic and non-economic processes is undeniable, globalization encompasses more than the redefinition of relationships between and among nation states, transnational corporations, and international organizations, as both critics and defenders of globalization often assert. Almost always left out of the analysis are the differences in the effect of these relationships on communities and other forms of local organization and more importantly, how those forces are shaped at the local level. The view that this paper proposes is that local actors are not necessarily relegated to the role of passive recipients of immutable global forces, and that the economic, social, and cultural impacts of globalization are not uniform among or within countries. Moreover, globalization has produced discontent as people and money are subjected to new patterns of mobility, while externally-imposed conditions are confronted by struggling nation states.

In other words, although much of the Third World still faces poverty and inequality [5], the impact of globalization is neither monolithic nor uniform, and local response is not only possible, but actually offers viable options to economic and political domination and cultural homogenization. In this view, for instance, local collective capacity in Ecuador continues to represent an effective counterweight to global forces such that globalization can, in effect, be shaped at the local level [6,7]. This is so in part because local culture remains a vital force despite homogenizing influences and can even be brought to bear in order to assert and reassert local values and practices [8]. More dramatic, perhaps, but no less relevant, is that the effects of globalization have been actively resisted throughout the world, including Latin America [9,10]. Local, regional, and national resistance to unpopular measures in Ecuador [11] has strengthened the indigenous movement as it confronts transnational capital so that grass roots democracy has been strengthened [12]. At the local level, for instance, public health can be put at the service of real people at the local level, and in addition, communities can and do participate in developing and implementing health care that meets their needs.

## Epidemiologic overlap: a global process

Just as economic, political, social, and cultural relationships are emerging throughout the world, patterns of morbidity and mortality are also undergoing complex patterns of epidemiologic transition that vary among and within countries [2]. But the particular path that epidemiological transition takes in a given case is closely related to social, economic, political, and cultural systems and processes that are, in turn, being redefined by globalization. Of particular relevance are the interrelationships among poverty, inequality, and health [13,14]. These interrelationships are particularly germane in contemporary Ecuador [15,16] and throughout Latin America [17-21].

The basic model of epidemiologic transition posits that mostly because of enhanced scientific understanding leading to the germ theory of disease and systematic improvements in sanitation infrastructure, four groups of what Omran [2-4] called "traditional" health problems began to recede in industrialized countries in the 19^th ^and early 20^th ^centuries: (1) communicable diseases, including respiratory illnesses and tuberculosis, diarrheal diseases, vaccine preventable diseases, and vector-borne diseases such as malaria and dengue; (2) poor health outcomes in mothers and infants related to reproduction and childbirth; (3) nutritional deficiencies; and (4) illnesses related to poor sanitation, especially water-borne pathogens in public water supplies and deficient sewage disposal. These problems are exacerbated by health care systems that lack the resources and capacity to attend to more than the most basic health problems. According to the basic model, the "traditional" conditions are gradually supplanted by a different set of "modern" health problems: (1) cardiovascular diseases, (2) malignancies due to cancer, (3) stress and other mental disorders, (4) diseases related to aging (such as Alzheimer's disease), (5) accidents (both traffic and occupational), and (6) emerging and re-emerging diseases and conditions, including overweight and obesity, diabetes, and hypertension. These conditions are exacerbated by health care delivery that is inadequate because of poor coverage, urban bias, limited outreach, poorly trained health care professionals, overly centralized operation, and an emphasis on curative rather than preventive care [3,4].

The conception of the epidemiologic transition represents less a theoretical construct than a descriptive model, which was not intended to be and should not be taken as an extension of modernization theory as postulated beginning in the 1960s [22], according to which, development is thought of as a series of stages through which all societies pass [23]. Rather, the model describes a variety of global and national processes that have shaped the evolution of health conditions throughout the world and in different historical moments. The simultaneous expression of morbidity and mortality due to "traditional" and "modern" health conditions obliges us to reevaluate the basic model of epidemiologic transition in light of diverse social and economic conditions. First, "traditional" diseases have not disappeared from industrialized countries, and a panoply of new and re-emerging infectious diseases pose new threats. Second, underdeveloped countries like Ecuador continue to experience high prevalence rates of infectious and communicable diseases, but at the same time, increasing rates of chronic and non-transmissible diseases associated with later phases of the epidemiologic transition [24]. Consequently, on one hand, well-documented general trends in global public health can be observed. For example, chronic diseases now account for 59 percent of the 57 million deaths reported worldwide (about half of these attributable to cardiovascular disease) and 46 percent of the global burden of disease [25]. At the same time, though, chronic diseases have become increasingly prevalent in underdeveloped countries and less prevalent in industrialized countries. On the other hand, traditional health problems in the former remain highly prevalent. For example, about 60 percent of all deaths among children under the age of five in the world are associated with malnutrition, and Vitamin A and iodine deficiencies continue to take heavy tolls in underdeveloped countries [26].

In other words, evolving health profiles in industrialized and underdeveloped countries suggest that the epidemiologic transition involves more than the gradual replacement of one set of diseases with another and that the epidemiologic transition can be more accurately described as a double epidemiologic overlap, one internal and one global [27]. The first overlap is represented by the continued high prevalence rates of both "traditional" and "modern" diseases in countries like Ecuador. But the burden of disease (which includes mortality and morbidity) is not uniformly distributed within the population. Rather, differences within countries can be attributed to inequalities related to socioeconomic factors such as income, occupation, ethnicity, level of education, and rural/urban residence. The second overlap comes about because as a product of globalization, the health profile of different groups of residents in underdeveloped and industrialized countries overlap. In both cases, the wealthy experience relatively lower rates of disease because of access to globalized health services (within or outside their own borders), information, healthy diets, and protection from environmental and occupational risks. At the same time, the rural and urban poor in both cases experience higher rates of both traditional communicable and infectious diseases (many of which are related to poor sanitary conditions, unhealthy housing, and ineffective control of vectors) and modern diseases, which are exacerbated by limited access to health care and failed health care policies.

The second overlap is a product of increasing integration into global markets, for example, in the production and processing of export-oriented agricultural commodities (much of it involving non-traditional products like cut flowers, tropical fruit, and temperate vegetables). This process connects the rural and urban poor in Ecuador (whose own consumption consists of increasingly more processed foods of poor nutritional quality) with new forms participation in global supermarkets by residents of the industrialized countries [28]. But consumption patterns vary within populations: those typical of the tiny affluent elite in Ecuador are similar to those of their northern counterparts–but in a lagged fashion. Among the imported consumer items available at high cost in elite supermarkets in urban Ecuador are imported processed, canned, and frozen items. These items represent a unique form of prestigious consumption because they reflect the same kind of expensive, flexible, and niche-driven consumption in industrialized countries. Moreover, among the Ecuadorian elites, health behaviors and health status now approximate patterns found in the industrialized countries. This is not a coincidence, because these segments have the same level of health care, which is secured (and often paid for through private insurance) either in local, private clinics and hospitals that are indistinguishable from those in industrialized nations, or in facilities actually located in the industrialized countries, especially in the southern United States.

The epidemiologic transition model proposed by Omran [4] takes into account these complexities and variations, which are found among and within countries. Thus, the "western" variation experienced by the presently industrialized countries has played out in five stages: (1) an age of pestilence and famine that occurred through the early 19^th ^century; (2) an age of receding pandemics beginning in the 19^th ^and early 20^th ^centuries; (3) an age of increasing degenerative, stress and man-made conditions that is still underway in some places and populations; (4) an age of declining cardiovascular mortality, ageing, lifestyle modifications, and emerging and resurgent diseases, now clearly observable in the United Stages and other industrialized countries; and (5) a future stage of "aspired quality of life, with paradoxical longevity and persistent inequalities" [[4]: 102]. This analysis also points out that contemporary social structures in the western transition model are characterized by generally improved living conditions, improved sanitation, small family size, and enhanced education and participation among women; while curative and preventive health care is organized at national and subnational levels and health insurance is available for individuals, groups (via employment and managed care plans) and entire nations (as in Great Britain). On the other hand, during the fourth stage of the transition, some residents of industrialized countries may experience limited access to health care, increased cost, and over-specialization of health services [[4]: 104].

In contrast, countries in Latin America and the Caribbean have followed a different, non-western model; for example, Ecuador, Peru, Paraguay, and the Dominican Republic typify the "lower intermediate" variation of the non-western model. According to this model, countries like Ecuador experienced the traditional diseases described above in the early 20^th ^century (until about 1940), when they began the process of epidemiologic transition, followed by epidemiologic overlap. The co-existence of traditional and modern health conditions is compounded by poor health care because of health systems and medical training that function poorly in the face of multiple new demands. This "triple health burden" [[4]: 106] distinguishes the epidemiologic transition in countries like Ecuador from that in countries like the United States [15,16,20,21].

## Ecuador: globalization and health as poverty and inequality

Ecuador's role in the global economy is very small; its GDP of about 19 billion dollars amounts to less than one tenth of Wal-Mart's annual sales. Nevertheless, Ecuador is still intimately linked to processes of globalization in at least six ways. First, transnational companies (including the two largest banks in the world, Citibank and Bank of America) operate in Ecuador. Second, while Ecuador continues to export traditional commodities (especially oil, bananas, coffee, and cocoa), it has also aggressively embarked upon the export of non-traditional products, mostly agricultural–notably, cut flowers [29]. Third, Ecuadorian workers produce for a global market, both at home and as transnational migrants [30]. Fourth, it is signatory to the World Trade Organization's most recent agreements, which govern global trade and finance and is actively engaged in different regional trade agreements. Fifth, it is heavily indebted to transnational banking institutions and multilateral lenders, which have imposed strict conditions related to their loan portfolios. For instance, an agreement signed with the IMF in 2000 contained 167 loan conditions that involved, for example, the privatization of potable waters systems, a new oil pipeline contract, layoffs of some public employees and wage cuts for others, and increases in the price of basic commodities like cooking oil [31]. Sixth, while autochthonous culture remains vibrant, imported culture floods local markets in the form of language, food, dress, and music.

Insertion into the global economy does not occur in a domestic vacuum, though; Ecuador has experienced unprecedented social and demographic change in the past several decades, producing profound transformation in its social structure, as reflected in the contribution to total GDP by agriculture, industry, and services. (See Table 1.) Employment patterns have shifted in parallel fashion; only eight percent of the economically active population now works in agriculture, 24 percent in industry, and 68 percent in the service sector [32].

**Table 1:** Distribution of gross domestic product by sector. Ecuador, 1965–2004. Percent.

	Agriculture	Industry	Services
1965	27	22	50
1988	15	36	49
2004	7	40	63

SOURCE: [33:182; 34: 296].

These changes are closely associated with permanent rural-urban migration. Ecuadorian society was largely rural and agrarian through the mid-20^th ^century, but 63.2 percent of its population was urban in 2001, and the figure is projected to reach 69.4 percent by 2015. While Quito and Guayaquil have grown dramatically–largely because of rural-urban migration–small and intermediate cities have grown even more quickly in many cases. Urban growth in Ecuador is further fueled by cyclical and temporary immigration by the rural poor in order to supplement meager rural household income with sporadic or temporary incomes derived from the informal urban sector [35].

Problems related to rural poverty are generally not resolved by migration, though; they are merely urbanized. Thus, urban unemployment nearly doubled from 9.2 percent in March 1998 to 17 percent in July 1999 and only returned to 9.3 percent by December 2005. In addition, underemployment (mostly in informal microenterprises) stood at 49.2 percent at the end of 2005. Consequently, poverty and indigence (or extreme poverty) expanded beginning in 1990, as shown in Table 2, and levels remain essentially unchanged today. This trend mirrors stagnant and declining real wages, which have only recently risen above those of several decades ago [36,37].

**Table 2:** Poverty and Indigence in Ecuador, 1995–2001. Percent.

	**Poverty**				**Indigence**			
	**1995**	**1998**	**2000**	**2001**	**1995**	**1998**	**2000**	**2001**
**Rural**	75.8	82.0	84.1	77.5	33.9	46.1	58.2	50.5
**Urban**	42.4	48.6	60.3	51.6	10.6	13.0	30.3	24.7
**Total**	55.0	62.6	68.8	60.8	20.0	26.9	40.3	33.8

SOURCE: [37: 50].

Crisis-driven poverty is also reflected in the distribution of resources and consumption. As an agrarian society, Ecuador was historically characterized by concentrated land ownership. Today, inequality in an increasingly urban, service-driven society is reflected in income and living conditions. In 1988, the wealthiest quintile of the population earned 50.6 percent of total income, while the poorest quintile earned 3.9 percent. But in 2004, the gap was even wider: the wealthiest quintile earned 62.3 percent of the population, while the poorest quintile earned only 1.7 percent [36]. Not surprisingly, the Gini coefficient of income inequality increased from 0.49 in 1995 to 0.57 in 1999 and 0.62 in 2001 (following dollarization of the economy), returning to 0.42 in 2003. Similarly, the Gini coefficient of consumption inequality has changed little, decreasing from 0.41 in 1995 to 0.38 for 2003–2004 [38].

These differences are closely related to gaps in living conditions. For example, in 2000, 77 percent of the population in the wealthiest income decile had access to a private flush toilet, compared to only 12 percent of people in the poorest income decile. Similar patterns are observed when comparing urban to rural areas; in 2002, 80 percent of urban Ecuadorians had access to improved sanitation while only 59 percent of rural residents did [36]. Access to clean water is a fundamental aspect of public health, and Table 3 shows enormous breaches between rural and urban residents and between the wealthy (top decile) and poor (bottom decile).

**Table 3:** Access to a source of clean water. Ecuador, 1999 and 2002. Percent.

	**Poorest decile**	**Wealthiest decile**	**Total 1999**	**Total 2002**
**Urban**	56.2	90.8	75.3	92
**Rural**	42.3	49.1	46.3	77
**Rural dispersed**	11.2	26.3	18.5	–

Source: [39:238].

Over a decade ago, poor living conditions were shown to be associated with adverse health outcomes among the poor in Ecuador [40]. Perhaps most dramatically, the ratio of the poor/non-poor risk of dying is more than 4 to 1 for Ecuadorian women and almost 3 to 1 for men [[41]: Statistical annex, table 7]. Gaps between urban and rural residents and by level of educational attainment further illustrate these relationships. Table 4 provides data on two sensitive indicators of health and development and suggests that substantial gaps in health outcomes remain, based on rural/urban residence, level of education, and province of residence (which reflects, among other things, race and ethnicity).

**Table 4:** Health and development disparities, Ecuador. Rates of fertility and infant mortality.

	**Urban areas**	**Rural areas**	**No education or primary**	**Secondary education or more**	**Lowest provincial rate**	**Highest provincial rate**
Fertility rate per woman 15–49	2.8	4.3	5.6	1.9	2.7	4.7
Infant mortality rate per 1,000 live births	22.0	40.0	51.0	11.0	26.0	34.0

SOURCE: [39: 241].

Health inequalities, understood as gaps in both access to care and outcomes, were exemplified by the rapid spread of cholera in 1991 from the port city of Callao, Peru through virtually the entire continent. Cholera struck almost exclusively in urban neighborhoods and poor rural communities, where morbidity and mortality were due to unsafe drinking water and inadequate sanitation, as well as consumption of unwashed or uncooked foodstuffs [42] and lack of timely and effective treatment. After Peru, Ecuador had the highest prevalence rate (450.9 per 100,000) and the most cases (46,284) in the first year (1991), and total cases exceeded 93,000 through 2000 [[39]: 310–311]. But exposure, morbidity, and mortality due to the disease were unevenly distributed: the poorest neighborhoods, particularly on the Coast, were heavily affected, while populations with access to safe supplies of treated public water were not. Cholera was present in relatively isolated highland indigenous communities, where mortality rates due to the disease were six times the national average [43].

## Epidemiologic overlap in Ecuador

Table 5 reflects the evolution of causes of death in Ecuador. It can be seen that of the 15 leading causes of death, nine (other heart disease, cerebrovascular diseases, diabetes mellitus, hypertensive diseases, aggression, isquemic heart disease, traffic accidents, malignant tumors, and self-inflicted injuries) can be classified as modern conditions. It can be noted in passing that the prominence of the "other heart disease" category has two explanations. First, as the population gradually ages and enters the final stages of the epidemiologic transition, heart disease will become more prevalent. Second, however, this particular cause of death is often ascribed when accurate information is lacking, particularly when people die of causes that are either poorly treated or not treated at all, when no autopsy is conducted, and when underlying causes leading to heart failure are never established.

**Table 5:** Principal causes of death. Ecuador, 2004 (per 10,000 inhabitants).

**Cause**	**Total**	**Males**	**Females**
1. Other heart disease	3.1	3.1	3.1
2. Pneumonia	2.3	2.5	2.1
3. Cerebrovascular diseases	2.3	2.4	2.2
4. Diabetes Mellitus	2.1	1.8	2.3
5. Hypertensive diseases	1.9	2.0	1.8
6. Aggression	1.8	3.2	0.3
7. Isquemic Heart Disease	1.8	2.1	1.4
8. Perinatal infections	1.5	1.7	1.2
9. Traffic accidents	1.4	2.2	0.7
10. Liver diseases	1.3	1.7	0.8
11. Malignant tumors, stomach	1.1	1.3	1.0
12. Chronic lower respiratory infections	0.7	0.8	0.6
13. Self-inflicted injuries	0.6	0.9	0.4
14. Septicemia	0.5	0.5	0.5
15. Respiratory tuberculosis	0.5	0.7	0.3

SOURCE: [44].

It should be noted that the epidemiologic transition in Ecuador occurred in the context of generally improved health outcomes, as measured by classic indicators; life expectancy at birth increased from 58.8 years (1970–1975) to 70.8 years in the 2000–2005 period, the infant mortality rate decreased from 87 per 1,000 live births in 1970 to 24 in 2001, and measles vaccination rates for one-year-olds increased from only 60 percent as recently as 1990 to 99 percent in 2001. Many of the changes are related to the gradual aging of the population; while 4.9 percent of Ecuadorians were over the age of 65 in 2001, the projection for 2015 is 6.6 percent. These are relatively low proportions (that of Uruguay is more than twice that of Ecuador), but it portends an important change in the future, as the presently bottom-heavy age pyramid gradually shifts upward.

The effects of the epidemiologic transition in Ecuador can also be seen in Table 6, which provides data on morbidity as measured through hospital discharges. While it is true that these data probably underestimate less serious illnesses that do not require attention at a hospital (or for which many poor people would be unwilling or unable to pay), they nevertheless portray the relative contribution to the total burden of disease in the country. The poor state of health among Ecuadorian women is reflected by the fact that conditions related to pregnancy and child birth represent the top three causes of morbidity for men and women, and about 18 percent of the total. Panel A also shows that at least nine of the top ten causes of morbidity are traditional conditions that would be observed in the earlier stages of the epidemiologic transition. (Attributing fractures as a cause of morbidity to either the traditional or modern category is problematic).

**Table 6:** Principal causes of morbidity: hospital discharges, Ecuador, 2003. Rates per 10,000 inhabitants.

**A. Total**	Rate
1. Other complications from pregnancy and birth	10.4
2. Other pregnancies terminating in abortion	4.2
3. Other maternal conditions related to the fetus, amniotic cavity, and possible problems with birth	3.4
4. Diarrhea and gastroenteritis, presumably infectious	3.2
5. Colelitiasis and colecystitis	2.9
6. Pneumonia	2.7
7. Other traumas	2.5
8. Diseases of the appendix	2.3
9. Fractures	1.4
10. Other infectious intestinal diseases	1.3

**B. Males**	

1. Other traumas	5.7
2. Diarrhea and gastroenteritis, presumably infectious	5.5
3. Pneumonia	4.6
4. Diseases of the appendix	3.9
5. Hernia	3.2
6. Fractures	3.1
7. Colelitiasis and colecystitis	2.6
8. Hyperplasia of the prostate	2.1
9. Other infectious intestinal diseases	2.1
10. Other respiratory problems in the perinatal period	2.0

**C. Females**	

1. Other complications from pregnancy and birth	15.1
2. Other pregnancies terminating in abortion	6.1
3. Other maternal conditions related to the fetus, amniotic cavity, and possible problems with birth	4.9
4. Colelitiasis and colecystitis	3.1
5. Diarrhea and gastroenteritis, presumably infectious	2.3
6. Pneumonia	1.9
7. Diseases of the appendix	1.7
8. Mioma of the uterus	1.3
9. Diabetes mellitus	1.1
10. Other problems of the urinary tract	1.1

SOURCE: [44].

Data disaggregated by gender reveal that diabetes appears as an important cause of morbidity in women. In addition, the "other heart conditions" category probably represents further underestimates of chronic disease prevalence, including diabetes, which is asymptomatic in its early stages. (At the same time, screening for diabetes among asymptomatic persons at potential risk is nearly inexistent in Ecuador.) Moreover, diabetes is closely associated with overweight and obesity, which is increasing in Ecuador because of changing socioeconomic conditions related to urbanization, occupational structure, diet, and physical activity. Similarly, among men, conditions of the prostate appear as a leading cause of morbidity in Ecuador. This category probably signals increasing prevalence of cancer in men and women. Prevalence data for cancer is incomplete at best, since services of screening and early detection are rarely available to the bulk of the population.

General improvements in the indicators of public health and changing patterns of morbidity and mortality were not equally distributed within the population, however. Several studies confirm that health conditions vary by social group within the population. Regarding "traditional" health conditions:

• A national survey conducted in the mid-1980s found significant differences among social classes in the prevalence of infant and child malnutrition [45]. More recent studies confirm that these differences persist [15,43,46], and nationwide data for 2004 clearly demonstrate that chronic malnutrition (stunting) in children is closely related to poverty, residence in rural and highland areas, and indigenous ethnicity [47].

• Vitamin A deficiency continues to place some segments of the population at risk, particularly households in the highlands, indigenous households, rural households, and households in which the mother has no formal education or in which children are underweight or stunted [48].

• Chagas disease, a preventable vector-borne disease, is endemic in the Oriente and in the Guayas River basin. Between 120,000 and 200,000 Ecuadorians are infected and between 2.2 and 3.8 million live under the risk of transmission of the disease [49].

On the other hand, the "modern" health problems identified by Omran are highly prevalent [2-4].

• The prevalence of overweight and obesity is now an epidemic only recently recognized. As of 2004, 40.4 percent of women were overweight (BMI of between 25 and 29.9) and 14.1 percent were obese (BMI over 29.9). At greatest risk are the urban poor because of factors associated with urbanization including changing diets, lifestyles, and occupational structure [50-52]. Overweight and obesity represents a critical feature of public health because it is associated with diabetes, heart disease, hypertension, and some forms of cancer [47].

• A study of the rural area around Borbón on the northwest coast found that cardiovascular diseases were the primary cause of death among adults, and that arterial hypertension, which was uncontrolled in most cases, was a major cause of mortality [53].

The situation of cancer merits special mention because it is not only an emerging disease in Ecuador, but because outcomes (both access to care and outcomes) reflect class-based differences. This is a particularly important factor in the case of diseases that may have low death rates when timely screening and treatment are available, but where death rates are high when early detection is not available. The few available studies reflect trends associated with cancer mortality rates.

• Uterine cancer has declined dramatically in industrialized countries, but more slowly in Latin America. Rates have changed little in Ecuador, however [54].

• Cancers related to occupational and environmental conditions pose additional risks for disease. For example, men and women who live around oil fields in the Amazonian provinces of Sucumbios, Orellana, Napo, and Pastaza face elevated risks of cancers of the stomach, rectum, skin, soft tissue, and kidney. In addition, women have increased risk of cancers of the cervix and lymph nodes; and children under the age of 10 have a higher risk of haematopoietic cancers [55].

• The age-adjusted incidence for cervical cancer is approximately 48 and mortality is approximately 19 per 100,000 [56]. This form of cancer is mainly associated with the human papilloma virus, but also to other factors, including poor diet, low life expectancy, barriers to health care, and low birth weight children. Protective factors include low fertility and delayed age at first childbirth. Incidence and mortality rates for cervical cancer also remain high (as compared to significant declines in urbanized countries) because of lack of prevention and control measures (particularly screening), which can reduce both mortality and incidence by 90 percent. Even when screening is available, inadequate collection and analysis of the samples and incomplete follow-up of women after testing further endangers poor women in particular. In sum, existing programs are "piecemeal, lack both organization and quality control, and have failed to meet their objectives" [56].

• While the prevalence of lung cancer is not particularly high, outcomes are poorer than expected because of the poor quality of care for those who are screened and treated; outpatient evaluation "is an efficient, slow, and potentially dangerous process in cases in which the probability of a cancer diagnosis is high" [[57]:167].

These data suggest that within Ecuador, the epidemiologic transition plays out differently among different populations, so that the non-western model displayed for the country as a whole must be interpreted as an essentially polarized variant in which particularly vulnerable segments of the population (rural, highland, indigenous and Afro-Ecuadorian, and the urban poor) continue to experience a protracted period of overlap.

In addition, part of the explanation the persistence of gaps in health outcomes lies in the Ecuadorian health care system. Despite important changes in the system in the past decade, the poor, including those who are either unemployed or the nearly half of the population who work in the informal sector (including peasant farmers), primarily use facilities operated by the Ministry of Health (MOH) while employees in the formal sector have access to facilities operated by the Social Security System (IESS). These facilities include rural health posts, regional hospitals that provide both ambulatory care and a limited number of beds, and larger tertiary hospitals. But the quality of service in public facilities has declined due to funding shortfalls. Moreover, the quality of care in MOH and IESS facilities is not the same; in rural areas, Social Security clinics provide better care than Ministry of Health clinics [58]. In either case, health care in the public sector is largely curative rather than preventive, and given poor living conditions and stagnant incomes, as well as the institution of user fees, most of the rural and urban poor are unlikely to be screened for cardiovascular conditions such as high blood pressure, those associated with overweight and obesity (especially diabetes), and cancers (such as prostate, cervical, and colorectal) that are largely asymptomatic until critical stages are reached.

Private facilities include modest local clinics that may be operated by a single physician, as well as state-of-the-art hospitals that provide roughly the same level of care as the best facilities in the world. Such facilities are largely accessible only to Ecuadorians who either have private insurance coverage or can pay the costs out-of-pocket.

## Local alternatives to epidemiologic overlap and globalization

Public spending for health care in Ecuador reflects the enormous gap between what is needed and what is actually provided. While health inequalities, understood in terms of access and outcomes, remain the hallmark of the Ecuadorian health care system, alternatives have been proposed and implemented at the local level. The rural poor are astute in their ability to assess the causes of poverty and realistic approaches to overcoming it [7]. Furthermore, as long practiced throughout Latin America, social medicine recognizes the multiple interrelationships between public health and socioeconomic conditions, critically assesses the "premise that societal arrangements of power and property powerfully shape the public's health," and acknowledges the role of external forces, especially the effects of "neoliberal economic policies, such as the North American Free Trade Agreement (NAFTA), which result in economic austerity plans, environmental degradation, and growing intra-and interregional disparities in health" [[59]: 1989]. Social medicine also includes a strong notion of social justice [60].

Local participation optimizes the likelihood of sustainability, particularly since experience shows that in Ecuador, community-based assessments and participation shift responsibility to the communities. The community-based approach represents a practical and viable alternative to planning, implementing, and evaluating actions that respond to local needs, especially in partnership with local NGOs and universities [61]. The importance of local control is officially recognized in Ecuador, which like many other countries has undertaken a process of decentralization supported by legislation and regulation. The basic tenet of this transformation is the assignation of responsibilities–and funds–to local and provincial jurisdictions. But not all local authorities have the capacity or experience to manage health systems and other sources of funds, especially taxes, are often lacking at the local level [62].

In spite of the obstacles, experiences in local planning and implementation of health care services–successful, partially successful, and even ultimately unsuccessful–suggest that alternatives to inefficient, centralized services may represent at least a partial solution that not only can successfully address pressing health problems, but also empower local populations.

With respect to financing, examples of decentralized health insurance include the following.

• While the national plan for universal health insurance has stagnated, local examples suggest that when local capacity and political will are present, health coverage can be enhanced substantially. In mid-2005, the Metropolitan District of Quito launched its Metropolitan Health Insurance program. Beginning with only 79 affiliates, the program had 5,000 July 2005 and 12,200 by January, 2006. The system has integrated existing groups as well as individuals and provides for services in 40 clinics. Affiliates pay $3.00 per month, for which they receive services up to a value of $1,000. Preventive care is provided, including prenatal care and growth monitoring of children under the age of five, as well as surgery, other curative care, and hospitalization. The goal of the program is to cover 25,000 by the end of 2006 out of a target population of 300,000 [63].

• In Guayaquil, the Program of Popular Insurance was inaugurated in January 2006 and in its first week covered 50,000 of 135,000 potential beneficiaries. It provides for health care in 45 centers [64].

• Community-based health insurance is combined with the provision of health services in subcenters (*Jambi Huasi*) in the provinces of Cotopaxi, Tungurahua, Cañar, Azuay, Pichincha, Guayas, and Napo. Support is provided by local and international NGOs, universities, multilateral organizations including the World Bank. One analysis [65] concludes that membership in prepaid health plans was limited, but that this system represents a potentially important vehicle for developing local capacity. An important aspect of the *Jambi Huasi *system is that it protects cultural and linguistic features of local communities by combining western and traditional medical treatment. For example, in the largely indigenous town of Otavalo, nearly 10,000 people had used the *Jambi Huasi *services by 1998, and about half used traditional healers. Quechua-language services provided in the clinic and in the field increased awareness of reproductive health issues, with the result that contraceptive rate increased from 10 percent to 40 percent, while both infant and maternal mortality rates declined [66].

• A decentralized, private health plan was less successful. The Pedro Vicente Maldonado Hospital, located in the semitropical region of western Pichincha Province, offered low-cost, prepaid health insurance. For thirty dollars per year, adults could receive five consultations, two emergency room visits, seven days of hospitalization, a 25 percent discount in the cost of surgery, all prenatal exams, all costs related to childbirth, care for newborns, two dental visits, preventive care for diabetes and hypertension, a 50 percent discount in the purchase of medicines, a 50 percent discount in the cost of X-rays, a 25 percent discount on all exams, all costs related to the treatment of snake bite and related to stabilizing traumas, and a 50 percent discount in ambulance fees. A similar program was available to children for an annual fee of 15 dollars. This plan ultimately failed, though, because few local residents enrolled in the plan.

Examples of local health systems and local participation in addressing specific health problems also suggest that response at this level is a viable alternative:

• Under the leadership of an indigenous mayor (now nationally prominent) a collective approach to public health in the northern highland town of Cotacachi began with the formation of a broad-based health committee in 1996. A commission with representation from the public health and education sectors as well as local organizations planned a health survey, trained interviewers, and conducted a diagnostic survey based on problems identified by the community. Cotacachi has since developed its own plan to meet the Millennium Development Goals [67].

• Community health campaigns supported by public and private alliances are increasingly common. For example, in Cotacachi, a recent campaign supported by a local hospital, a local foundation, and local communities provided a variety of services (dental, preventive care for hypertension and other conditions, prenatal care, cancer screening, and vaccinations) to nearly 3,500 people [68].

• A community-based surveillance system was critical in eliminating yaws in Esmeraldas Province [69].

• A gender-based approach to community development has been employed to empower poor urban women in Guayaquil, including the establishment of their own health center [70].

## Summary

In the first years of the millennium, the Ecuadorian health care system is at a crossroads. From a policy perspective, it is apparent that the government is ill-prepared to assume the responsibility for planning and financing care for an expanding elderly population. In July 2004, pensioners instituted a hunger strike when demands for increases in payments were ignored, and when the government did react, its proposal was to increase the national sales tax. The pensioners took such extreme measures (there were 17 fatalities) because the majority receive less than 100 dollars per month, and a substantial portion receive less than 50 dollars a month.

The epidemiologic overlap places the country in a double bind; not only is the risk of infectious and communicable diseases inadequately addressed, but the opportunities for timely screening and treatment of chronic and non-transmissible diseases and other modern health problems are extremely limited. For example, the rates of cancer incidence and mortality present challenges that can only grow in the future. First, it is difficult to interpret existing data. Rates of morbidity and mortality associated with different forms of cancer, for example, are almost certainly underestimated due to low levels of screening and correct diagnosis. Particularly among the poor, it seems very likely that a high proportion of cases go undetected. Second, even when cancer is detected, barriers to care (whether economic, cultural, or logistical) mean that a high proportion of cases very likely present at advanced or aggressive stages of the disease, meaning that rates of survival would be lower than expected. Studies in the United States reveal that barriers to care have just that effect among Latinos [71] even when objectively, screening and treatment services are much more widely available than in Ecuador.

In human terms, this means that in Ecuador and elsewhere in the Third World (among the poor in particular) men and women are sick and even dying without knowledge of their conditions and without access to even the most rudimentary screening and treatment services. Many forms of cancer are relatively easily treated in their early stages, but in many of these forms (including cervical, colorectal, and prostate cancers) early stages are asymptomatic. More effective screening programs are required, especially since as the population continues to age, prevalence rates can be expected to rise.

Nevertheless, the major obstacles to effective screening programs for cancer, cardiovascular disease, diabetes, and other "modern" conditions are poverty and inequality, which are problems that globalization does not address, and to the extent that attention is paid to economic and financial integration through enhancing the export sector, the effects may even be negative.

Fortunately, there is a long history and tradition of social participation in Ecuador that represent the potential basis for shaping the forces of both globalization and epidemiologic overlap. It is interesting to note that demands for decentralization as the most appropriate public policy response (in health and other sectors), come from both sides of the political spectrum. This unusual convergence is more apparent than real, though, since conservative models of decentralization focus on weakening the State while participatory, community-based alternatives are based on democratic principles of local participation as well as efficiency and effectiveness [72]. The removal of three successive democratically-elected presidents (Mahaud, Bucaram, and Guitierrez) in less than seven years only exacerbated systems of political patronage that have impeded the development of a coherent approach to the challenges presented by epidemiologic transition and overlap within the broader context of globalization.

Local control of health care is by no means a panacea. Economies of scale are limited or absent, and human resources are unevenly distributed. Local authorities are not by definition more committed to listening to local voices or addressing local needs (or less corrupt) than national authorities. Nevertheless, their ability to shape national policy (for example in participating in global alliances for solving health problems) and organize services represents a viable alternative.

Important challenges lie ahead. For example, few countries in the world have adequately addressed looming problems associated with modern health conditions. In the coming years, diabetes and related conditions will become so prevalent that it will no longer be possible to ignore them. In addition to the conditions mentioned above, those related to aging, such as Alzheimer's disease–and mental conditions in all age groups–will also be of increasing concern. The ability of local authorities (probably in new alliances with international organizations, national authorities, and even the private sector) to deal with these problems will be a major concern in the mid-21^st ^century.

## Competing interests

The author(s) declares that he has no competing interests.
